# Metal-Organic Frameworks With Variable Valence Metal-Photoactive Components: Emerging Platform for Volatile Organic Compounds Photocatalytic Degradation

**DOI:** 10.3389/fchem.2021.749839

**Published:** 2021-11-15

**Authors:** Yuhang Qian, Dongge Ma, Junbo Zhong

**Affiliations:** ^1^ Department of Chemistry, College of Chemistry and Materials Engineering, Beijing Technology and Business University, Beijing, China; ^2^ College of Chemical Engineering, Sichuan University of Science and Engineering, Zigong, China

**Keywords:** MOFs, photocatalytic degradation, indoor-air pollutants, VOCs, photocatalysis mechanism

## Abstract

With their outstanding diversities in both structures and performances, newly emerging metal-organic frameworks (MOFs) materials are considered to be the most promising artificial catalysts to meet multiple challenges in the fields of energy and environment. Especially in absorption and conversion of solar energy, a variety of MOFs can be readily designed to cover and harvest the sun irradiation of ultraviolet (UV), visible and near-infrared region through tuning both organic linkers and metal nodes to create optimal photocatalytic efficiency. Nowadays, a variety of MOFs were successfully synthesized as powerful photocatalysts for important redox reactions such as water-splitting, CO_2_ reduction and aqueous environmental pollutants detoxification. MOFs applications in indoor-air VOCs pollutants cleaning, however, are less concerned partially because of limited diffusion of both gaseous pollutant molecules and photo-induced active species in very porous MOFs structures. In this mini-review, we focus on the major breakthroughs of MOFs as photocatalysts for the effective removal of indoor-air VOCs such as aldehydes, aromatics and short-chain alcohols. According to their nature of photoactive centers, herein MOFs photocatalysts are divided into two categories to comment, that is, MOFs with variable valence metal nodes as direct photoactive centers and MOFs with non-variable valence metal nodes but after combining other photoactive variable valence metal centers as excellent concentrated and concerted electron-transfer materials. The mechanisms and current challenges of the photocatalytic degradation of indoor-air VOC pollutants by these MOFs will be discussed as deeply as possible.

## Introduction

With the increasing economic and industrial developments, more and more anthropogenic gaseous pollutants such as NO_x_, SO_2_, VOCs (volatile organic compounds), and PMs (particulate matters) are being emitted from exhaust gases of automobiles, power plants, steel mills, burning of straw and various other industrial sources (Poschl and Shiraiwa, 2015). These substances have been evidenced to lead to multiple threats to human health (Bari and Kindzierski, 2018), particularly in the confined space such as cabins, offices, hospitals and submarines. Among the various gaseous pollutants, VOCs are considered to be one of the most common and notorious pollutants of indoor air. VOCs are defined as those organic compounds with the normal boiling point between room temperature to 260°C. The common VOCs in indoor-air mainly include aromatics (benzene, toluene, xylene, mesitylene and styrene), short-chain alcohols (methanol, ethanol and isopropanol), short-chain carbonyl compounds (formaldehyde, acetaldehyde and acetones) and low molecular-weight aliphatic hydrocarbons (n-hexane, cyclohexane, and isoprene). (Li and Gaillard, 2009). Due to its high mutagenic and carcinogenic toxicity, VOCs in indoor-air are extremely hazardous to human health (Chen et al., 2009; Huang et al., 2014a). Now, more and more strict laws and regulations with lower limit of these indoor-air pollutants are issuing (Liotta, 2010).

To meet the demands of these pacts, various methods such as adsorption (Glomb et al., 2017), filtering (Koo et al., 2018), catalytic oxidation (Huang et al., 2015; Chen et al., 2020) and photocatalytic oxidation (Li et al., 2019) are emerging. Among these methods, photocatalysis is deemed to be one of the most promising technologies because it adopts the clean solar light (or indoor light) to drive the chemical degradation reactions. Since Fujishima and Honda reported that TiO_2_ can catalyze the water-splitting to generate H_2_ and O_2_ upon UV light irradiation (Fujishima and Honda, 1972), novel materials possessing photocatalytic activity are appearing at an exponential speed, especially in the past 2 decades (Ma et al., 2018; Ma et al., 2019; Li et al., 2020a). Especially, Yaghi’s group discovered the first metal-organic framework MOF-5, which is typically porous crystalline material and constructed from Zn^2+^ and H_2_BDC (1,4-benzenedicarboxylate) (Li et al., 1999). Garcia et al. thereafter evidenced its photocatalytic activity by the degradation of dye in water (Alvaro et al., 2007). From then on, a large number of MOFs-based photocatalysts have been prepared for the purpose of environmental pollutants degradation (Wang et al., 2014; Tian et al., 2018; Chen et al., 2019; Cheng and Zhang, 2020; Wang et al., 2020a; Han et al., 2021). However, compared with most researches focusing on the degradation of aqueous environment pollutants, less concentration has been paid to the gaseous pollutants degradation and elimination (Wen et al., 2019), especially indoor-air pollutants decontamination with MOFs photocatalysts. One of the main reasons is the restricted lighting-area and difficult diffusion of trace VOCs as well as their degradation products in the inconvenient packed gaseous-solid reactors, which makes few photocatalysts including most MOFs for such tough burdens. Nevertheless, some MOFs photocatalytic applications for indoor-air quality control have garnered considerable success albeit mostly in the lab scale. These achievements may shed light on the key molecular basis of MOFs photocatalysts for practicable degradation of indoor-air pollutants (Li et al., 2019).

In this mini-review, we will focus on two kinds of MOFs photocatalysts for typical indoor air VOCs treatments according to whether the metal nodes in MOFs are or not photoactive centers. For the former, since MOFs materials bear variable valence metal ion such as Ti^4+/3+^, Fe^3+/2+^, Cu^2+/1+^ as nodes, they act as photo-induced redox sites by themselves directly to initiate indoor VOCs degradation. For the latter, because MOFs are made of non-variable valence metal such as Zr^4+^, Zn^2+^ ion as nodes, they must combine other photoactive centers in which MOFs themselves act as excellent concentrated and concerted ET role to enhance photo-degradation. Some paragon examples of these two kinds of MOFs photocatalysts applied in decomposition of representative indoor air VOCs will be discussed in detail. The motivation of this mini-review is based on MOFs photocatalytic materials recently emerging of the outstanding diversities in both porous structures and performances that may just meet two challenges, namely, the restricted lighting-area and difficult diffusion of trace VOCs in the elimination of indoor air pollutants. To demonstrate and comment on photocatalytic removal of indoor air pollutants in terms of metal node redox properties of MOFs is the main innovation of this review.

## MOFs for Indoor VOCs Photocatalytic Degradation

### Basic Principles and Feature Advantages for MOFs Photocatalysis in Decomposing Indoor VOCs

**FIGURE 1:**
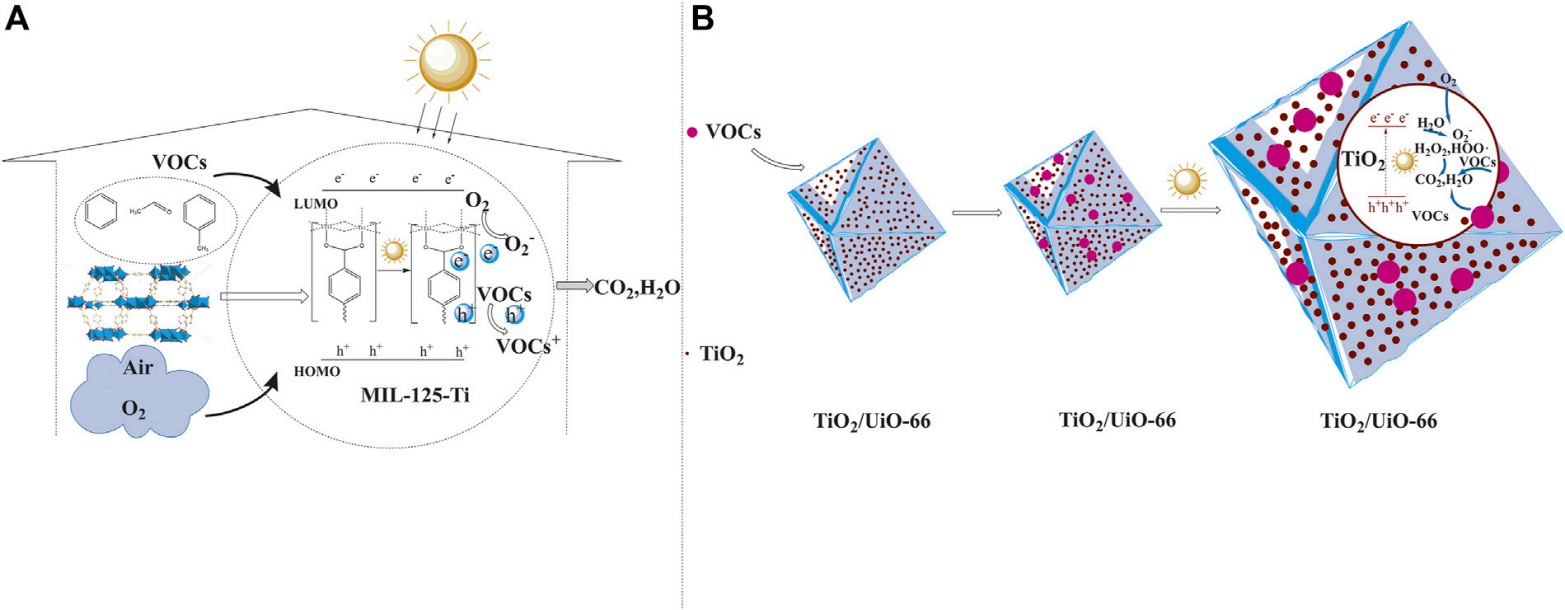
Schematic representation of MOFs as direct photoactive center such as Mil-125-Ti **(A)** or as base material to combine other photoactive component such as TiO_2_/UiO-66-Zr **(B)** for degradation of indoor-air VOCs.

Differing from the mature photocatalytic degradation of organic pollutants in water, the removal of indoor VOCs pollutants has its own characteristics. 1) Due to the gas-solid interface reaction traits, the highly effective oxidative degradation nearly for any organics by highly active ^•^OH radicals (generated via valence band holes, h_vb_^+^ + H_2_O → ^•^OH process) in traditional photocatalytic degradation of aqueous organic pollutants is invalid. Only the direct h_vb_^+^ oxidative degradation of VOCs is left behind (i.e., h_vb_^+^ + VOCs → VOCs^+•^). At this time, gaseous O_2_ will play pivotal roles: one is to rapidly capture, remove the conduction-band electron (e_cb_^−^) so as to efficiently generate more h_vb_^+^ to oxidize VOCs; another is directly adding to the VOCs^•^ intermediates (VOCs^•^ + O_2_ → VOCs-^•^OO), or the secondary products with e_cb_^−^ including O_2_^−•^, ^•^OOH, H_2_O_2_ by ET or the singlet ^1^O_2_ via energy transfer, which will diffuse in the pores and react with VOCs pollutants. The h_vb_^+^ cannot leave the surface to react with VOCs via indirect solvent-relay route, while O_2_ and its secondary active species can diffuse and migrate in MOFs channels. Thus, the design of MOFs structure will mainly focus on the O_2_ diffusion channels and favorable reaction sites (see Figure 1) (Li et al., 2019). 2) From the practical applications in indoor air cleaning, the as-used gas/solid photocatalysts hardly accommodate highly light-harvesting area/volume ratio whether photocatalysts are in membrane or powder forms. The optimum strategy to increase the efficiency of the surface photo-reaction is to utilize the convection and diffusion effect to concentrate and confine the VOCs in the outer-surface and internal pores of the whole catalyst. Compared with the previous common inorganic oxides and organic photocatalysts, MOFs possess large advantages on this occasion. Their abundant pores, large specific surfaces area (500–7,000 m^2^/g) can assist to pre-concentrate and store a large amount of VOCs. Once upon sunlight irradiation, the enriched VOCs can conveniently diffuse from the internal pores to the surface to be photo-degraded (see Figure 1A) (Saouma et al., 2018). 3) Combined the above-mentioned double properties of photo-degradation of indoor-air VOCs, the feasible MOFs photocatalysts are mainly divided into two categories. One is that the variable valence metal ions construct the MOF’s node, such as common Ti^4+^, Fe^3+^, Cu^2+^. They can convert to the low valence Ti^4+/3+^, Fe^3+/2+^, Cu^2+/1+^ upon irradiation by LMCT/MLCT mechanism, which creates the h_vb_^+^/e_cb_^−^ active center to react with VOCs and O_2_, respectively. Another kind of MOFs photocatalysts is using non-redox-active metal such as Zr^4+^, Zn^2+^ as the nodes and hybridizing with other photoactive materials (Ma et al., 2019). For these composite MOFs, the photoactive centers can be inorganic metal-oxides such as TiO_2_, ZnO, noble-metal nanoparticles and organometallic complexes. On this basis, MOFs mainly contribute to the large specific surface areas and abundant pore structures, which can pre-concentrate the indoor-VOCs and utilize its highly-ordered stacking π electrons to boost the composites photoactive center charge-separation efficiency (see Figure 1B). The critical point of this kind MOFs is the high accordance of the hybridizing interfaces and channels to the corresponding indoor VOCs. In the following part, we will divide these two categories of MOFs materials further into the most common Ti-based MOFs materials and other MOFs photocatalyst and, introduce their applications in the indoor-air VOCs photo-degradation.

### Ti-Based MOFs Photocatalysts for Degradation of Indoor Air VOCs

Due to the TiO_6_ octahedral coordination and Ti^4+^/Ti^3+^ redox similar to the star heterogeneous photocatalyst TiO_2_, Ti-based MOFs are investigated in depth for the purpose of the semiconductor photocatalysts for VOCs removal. By a microwave heating procedure, NH_2_-MIL-125 (Ti) was hybridized with graphene oxide (GO) to form a heterojunction photocatalyst (Li et al., 2018b). As-prepared 10-GO/NH_2_-MIL-125(Ti) sample exhibits much enhanced photo-degradation activity of gaseous acetaldehyde under visible-light irradiation (*λ* > 420 nm) in comparison with mechanically mixed two components and unmodified NH_2_-MIL-125(Ti). The authors attributed the superior photocatalytic performance of the hybridized photocatalyst to the enhanced visible-light absorbance, more electron carrier density, more efficient charge carrier transfer and less electron-hole recombination. This example indicates that the formation of heterojunction can considerably improve the MOFs photocatalyst performance in VOCs remediation.

Zhao et al. developed a N/Zn co-decoration strategy to strengthen the photocatalytic activity of MIL-125(Ti) towards indoor-air acetaldehyde oxidative degradation (Gao et al., 2020). N and Zn dopants were introduced by a simple solvothermal process. N/Zn co-doped MIL-125(Ti) exhibits 10.5 and 4.4 times higher removal rate of gaseous acetaldehyde under visible-light irradiation in comparison with Zn-doped MIL-125(Ti) and pristine MIL-125(Ti), even surpasses the state-of-the-art metal-oxides and noble-metal-based photocatalysts. DFT theoretical simulations evidenced the N/Zn co-doping tactic evolves the inter-gap band, which plays dual role either as shallow trap for charge carrier to enhance separation efficiency or narrowing the gap to increase visible-light absorptivity. This report shows that via the co-doping strategy to modify the surface acidity, polarity and the electronic structure, MOFs photocatalyst can achieve higher activity and humid environment resistance for VOCs removal.

The same group discovered that by an ionic-liquid assisted synthetic procedure, defect-rich polyaniline (PANI)/NH_2_-MIL-125(Ti) nanohybrid photocatalyst can realize ultra-rapid degradation of acetaldehyde even under high humidity (Shah et al., 2021). [EMIM]BF_4_ ionic-liquid was exploited to dissolve the PANI to oligomers and graft the *in-situ* formed PANI oligomers to NH_2_-MIL-125(Ti). The smart-grafted IL-PANI/NH_2_-MIL-125(Ti) exhibits strong interfacial π-π conjugation and van der Waals interactions between NH_2_-MIL-125(Ti) and PANI oligomer. Besides, the IL-treated PANI/interfacial displays more defects such as ligand vacancies and Ti^3+^ sites thereby created much enhanced Lewis acidity, which is critically important for effective adsorption of acetaldehyde with Lewis basicity (O lone pair). Interestingly, water molecules can enhance the performance of this photocatalyst. From the electron paramagnetic resonance (EPR) trapping experiments, H_2_O participates in ROS generation by reacting with O_2_^−•^ to yield more active hydroxyl radicals to oxidize acetaldehyde. The IL-treated PANI-Ti-MOFs exhibited much increased affinity to acetaldehyde and acetic acid in comparison with CO_2_, which improves the retention of the intermediate acetic acid in the photocatalyst reactive site and the desorption of the final innocuous CO_2_ product.

### Other Metal-Based MOFs Photocatalysts for Degradation of Indoor Air VOCs

Apart from MIL-125(Ti)-based MOFs materials, their Zr-MOF counterparts exhibit much enhanced stability towards humidity and long-term light-irradiation. Thus, it is also highly desirable to apply Zr-MOFs for photocatalytic VOCs degradation especially in highly humid environment. To just take advantage of the hydrolytic stability, if proper semiconductor or metal nanoparticles as photoactive centers were hybridized with Zr-MOFs, their photocatalytic performances can be considerably improved. By an *in-situ* solvothermal synthesis of NH_2_-UiO-66 (Zr) in the presence of pre-formed TiO_2_ nanoparticles, a TiO_2_/NH_2_-UiO-66 (Zr) composite photocatalyst was successfully prepared. (Yao et al., 2018). Ultra-small TiO_2_ nanoparticles were encapsulated inside the interior of Zr-MOF. The composite photocatalyst demonstrates much superior performance of the photocatalytic oxidation of styrene in comparison with TiO_2_ and pristine NH_2_-UiO-66 (Zr). The authors proposed that due to the affinity of styrene with Zr-MOF’s linker 2-aminoterephthalate by strong π-π interaction, the VOC (styrene) could be concentrated in the NH_2_-UiO-66 interior pores. The encapsulated TiO_2_ photoactive sites can contact more conveniently and react with the neighboring styrene inside the porous structure in comparison with styrene in the outer surface. Besides the easier diffusion and reaction of VOCs substrate, the photo-generated electrons and holes could be transferred and separated between NH_2_-UiO-66 (Zr) and encapsulated TiO_2_. Namely, the elaborate conjugation of semiconductors with stable Zr-MOFs significantly improves its photocatalytic activity for indoor VOCs degradation through concerted strong interfacial effects.

Besides common static VOCs photocatalytic degradation, continuous flow reactor system provides more potential for practical indoor-air decontamination. Hu et al. utilized an evaporation method generating TiO_2_-UiO-66-NH_2_ composite photocatalyst for gaseous toluene and acetaldehyde removal. (Zhang et al., 2020a). The large specific surface area of Zr-MOF considerably boosted photocatalytic performance of TiO_2_ under UV irradiation by providing better dispersion media for TiO_2_ nanoparticles. As-synthesized composite photocatalyst demonstrates exciting performance for toluene and acetaldehyde degradation with satisfactory conversion rate and CO_2_ selectivity. This demonstrates that smooth removal of indoor air VOCs by Zr-MOF-based hybrid materials can be realized even under very fast continuous flow condition.

Moreover, the core/shell NH_2_-UiO-66@TiO_2_ was also applied for the more effective photocatalytic degradation of VOC pollutant toluene. By the *in-situ* preparation of amorphous TiO_2_ from the hydrolysis of Ti (*t*-BuO)_4_ in the presence of pre-formed UiO-66-NH_2_, more effective photocatalytic removal of toluene can be achieved comparing with TiO_2_ and unmodified UiO-66-NH_2_. (Zhou et al., 2021). Detailed EPR mechanistic investigations were conducted compared with An’s and Hu’s pioneering work using the same type MOFs composite material, demonstrating that O_2_^−•^ superoxide anion plays a pivotal role during photocatalytic reaction.

To enhance the light-absorption and extend the absorption spectrum to the visible-light and near-infrared region, porphyrin-based organic linkers were introduced to the MOFs structure. After implantation of iron (III) ions into the center of porphyrin units of PCN-224 MOF photocatalyst bonded Zr-oxo cluster with *tetra*-kis (4-carboxyphenyl)-porphyrin, a novel Fe@PCN-224 photocatalyst was constructed (Shi et al., 2018). Its photocatalytic performance for oxidation of isopropanol (a common VOC pollutant of indoor air) into CO_2_ was significantly enhanced via Fe-implanted porphyrin-based Zr-MOF. Herein Fe not only improves the photo-generated charge-carrier separation efficiency, but also produces additional Fenton reaction activity to assist less active H_2_O_2_ transformation to much stronger ^•^OH by Fe^3+/2+^ cycles. This report provides an innovative strategy to combine Fenton reactivity with stable Zr-MOFs photocatalyst towards indoor air VOC pollutants such as isopropanol.

Zn-based zeolite imidazole framework ZIF-8 is another kind of highly stable MOFs against hydrolytic and photo-irradiation conditions (Huang et al., 2014b). To exploit its application for ethylene removal under UV irradiation, a bimetallic PtPd alloy encapsulated-ZIF-8 photocatalyst was prepared and exhibited synergistic function in contrast with the pristine ZIF-8 and monometal encapsulated ZIF-8 materials.

Although ZIF-8 photocatalyst can provide excellent performance under UV irradiation, it rarely has visible-light activity. To create its visible-light activity, a post-modification strategy was applied to fine-tune the chemical structure of the ZIF-8 photocatalyst.(Wang et al., 2020b). After heating in the air atmosphere, ZIF-8 was partially oxidized to incorporate an isocyanate group N=C=O, with the concomitant cleavage of N-methyl group of the pristine imidazole unit. The introduced isocyanate moiety not only strongly extends ZIF-8 absorption band-edge from 325 nm (UV) to 715 nm (visible light region), but also introduces more adsorption activity towards formaldehyde and less adsorptivity to CO_2_ comparing with its original ZIF-8 parent. Both the augmentation of light absorption, transmittance and formaldehyde adsorption result in much enhanced photocatalytic removal efficiency of indoor HCHO. Interestingly, water in this system can accelerate the generation of strongly oxidative H_2_O_2_ species thereby enhance the degradation of formaldehyde in moist air, by which further boosts its activity and stability for this porous photocatalyst under humid environment.

In addition, Cu and Fe-based HKUST-1 and NH_2_-MIL-101(Fe) MOFs also act as efficient photocatalyst for VOCs degradation under irradiation conditions. Cu-based HKUST-1 MOF material merged with anatase TiO_2_ semiconductor to form a core/shell structure with Cu_3_(BTC)_2_ (BTC = 1,3,5-benzenetricarboxylate) as the core and ultra-thin anatase film as a shell (Wang et al., 2016). The as-formed core/shell MOFs improve the efficiency of photo-generated electron-hole separation due to the facilitated ET from CB of TiO_2_ to HKUST-1 Cu^2+^ center. Moreover, the Cu-HKUST-1 core structure enhances the adsorption of isopropanol and the toxic intermediate acetone, while the final product CO_2_ is less adsorbed.

As an earth-abundant and non-toxic element, iron-based materials are considered as promising catalysts for remediation of indoor air. NH_2_-MIL-101(Fe) photocatalyst was reported to effectively remove the toluene in hermetic space (Zhang et al., 2016). The microspindle Fe-MOF photocatalyst demonstrates excellent toluene degradation efficiency upon visible-light irradiation because of the much intense and broad visible-light absorption spectrum. The NH_2_-terephthalate organic ligand acts as antenna to harvest visible-light to initiate LCCT (ligand-to-metal-cluster charge-transfer) process. Moreover, the Fe_3_-*μ*_3_-oxo cluster can directly absorb the visible-light and initiate an electron-hole separation event. Photo-induced electron and hole migrate separately to the catalyst surface to react with the adsorbed O_2_ and H_2_O molecules, yielding strongly oxidative ROS species such as O_2_^−•^ and ^•^OH to degrade toluene into CO_2_ and H_2_O by consecutive radical addition, hydroxylation, ring-opening and chain-cleavage processes.

### The Recent Progress in the Preparation of MOFs-Based Materials for the Elimination of VOCs

Great efforts have been devoted to developing MOF-based materials specifically towards photocatalytic elimination of indoor VOC pollutants in recent years. Every as-prepared MOF photocatalyst example commented in *Ti-Based MOFs Photocatalysts for Degradation of Indoor Air VOCs* and *Other Metal-Based MOFs Photocatalysts for Degradation of Indoor Air VOCs* section is now summarized in Table 1. More importantly, the latest preparation and modification strategies of MOFs-based photocatalysts are emerging, mainly for overcoming the inherent disadvantages in degradation of VOC pollutants (Hao et al., 2020; Li et al., 2020b; Zhang et al., 2020b). 1) The organic linkers with huge diversity in MOFs usually serve as antennas for light harvesting. Therefore, a preferred methodology has been attempted to improve the light absorption of MOFs by furnishing aromatic carboxylate ligands with −NH_2_ to improve its light harvesting (Fu et al., 2012). Indeed, most MOFs photocatalyst examples in Table 1 have -NH_2_ groups. 2) Location of the Pt and metalloporphyrin cocatalyst in MOF composites can significantly boost electron−hole separation (Xiao et al., 2016). In addition, noble metals loaded in MOFs commonly catalyzed VOCs molecules transformation even under mild conditions. 3) Combination of plasmonic metals and Schottky junction into MOFs can simultaneously improve light harvesting and charge separation (Xiao et al., 2018). Alternatively, integrating upconversion nanoparticles into MOFs photocatalyst is specially adapted to the use of indoor light (Li et al., 2018a). 4) Photothermal effect by both plasmonic metals and MOFs in MOF-based composites, such as PCN-224 (M) and Pt nanocrystals, is able to activate O_2_ to ^1^O_2_ under light irradiation, which ROS species driven by photothermal effect is very effective to selectively react with olefins VOC pollutants (Chen et al., 2017). All of these approaches to preparation and modification of MOFs photocatalysts show that the well-defined and tailorable MOF structures hold great advantages in eventually solving the problem of indoor VOC pollutants (Xiao and Jiang, 2019).

**TABLE 1:** Current state-of-the-art MOFs photocatalysts applied for VOCs degradation.

MOFs	VOC pollutant	C_VOC_	m_MOF_	Irradiation conditions	Degradation rate	Reference
GO/NH_2_-MIL-125(Ti)	acetaldehyde	1.95 mg/L	50 mg	300 W Xenon lamp with 420 nm filter	65%	Li et al. (2018b)
Mil-125(N-Ti_9_Zn_1_)	acetaldehyde	200 ppm	100 mg	300 W Xenon lamp with 420 nm filter	98%	Gao et al. (2020)
IL-PANI-NH_2_-MIL-125(Ti)	acetaldehyde	300 ppm	40 mg	300 W Xenon lamp full spectrum	92%	Shah et al. (2021)
TiO_2_/NH_2_-UiO-66(Zr)	styrene	30 ppmv with 35 ml/min	100 mg	300 W Xenon lamp full spectrum	99%	Yao et al. (2018)
TiO_2_/NH_2_-UiO-66(Zr)	toluene/acetaldehyde	25/30 ppm	100 mg	A 125 W high pressure mercury lamp UV light	73%/71%	Zhang et al. (2020a)
NH_2_-UiO-66-Zr@TiO_2_	toluene	150 ppm	100 mg	300 W Xenon lamp with 420 nm cut-off filter	77%	Zhou et al. (2021)
Fe^3+^/PCN-224	isopropanol	NA	50 mg	300 W Xenon lamp with 400 nm filter	NA	Shi et al. (2018)
Pt_5_Pd_5_@ZIF-8(Zn)	ethylene	100 ppm	200 mg	300 W Xenon lamp full spectrum	93%	Huang et al. (2014b)
ZIF-8-T	formaldehyde	20 ppm	50 mg	300 W Xenon lamp with 420 nm filter	100%	Wang et al. (2020b)
HKUST-1-Cu@TiO_2_	isopropanol	NA	NA	AM 1.5G simulator	NA	Wang et al. (2016)
NH_2_-MIL-101(Fe)	toluene	4 μl	20 mg	500 W Xenon lamp with 400 nm filter	79%	Zhang et al. (2016)

## Perspectives on Current Challenges of Elimination of Indoor VOC Pollutants by MOF Photocatalysis

Despite some successful applications of MOFs photocatalysts for the removal of indoor-air VOCs pollutants (Table 1), there still remain various challenges that decisively limit the practical application beyond laboratory level. Firstly, the considerably low space-time yields still restrain the large-scale treatment of real indoor air. Secondly, the mineralization yields are still not satisfactory. The common MOFs-based photocatalyst system often possesses less oxidativity compared with traditional UV-excitable metal oxides photocatalysts such as TiO_2_. Such poor mineralization results in the accumulation of organic intermediates on the surface of photocatalysts, which usually leads to catalyst deactivation and blocks the catalytic cycle. Although there have been some MOFs-based photocatalysts with excellent stability for moisture under long-term illumination, the weak coordinative bonds still restrain the discoveries of more powerful MOFs-based photocatalysts for VOCs removal. Lastly, up till now, there still lacks MOFs photocatalysts to realize indoor VOCs removal under NIR irradiation due to the low-energy of NIR photon not enough cleaving the high-energy VOCs chemical bonds. This considerably decreases the solar-light-utilization efficiency as NIR possesses about half of the overall energy of sunlight. The doping, co-doping, dye-sensitization can be useful methods to extend absorption spectrum and enhance charge-carrier separation. The utilization of the organic ligands with electron donor-acceptor structure is another potential tactic to enhance the visible-light absorption or even to induce NIR absorptivity. The merging of two-photon absorbing organic moiety may initiate its NIR photocatalytic ability for indoor VOCs degradation (Li et al., 2018a). Although great challenges facing to us in this research area, we believe that the future of MOFs photocatalytic applications for indoor VOCs degradation will be much exciting and awarding.
